# The disappearance of femoral head and neck resulting from extensive bone defect caused by secondary syphilis: a case report and literature review

**DOI:** 10.1186/s12891-018-2152-1

**Published:** 2018-07-25

**Authors:** Xiao Liang, Tang Liu, Chuang Yuan, Wanchun Wang, Peixiong Liang

**Affiliations:** 1 Department of Orthopaedics, the Second Xiangya Hospital, Central South University, 139 Renmin Road, Changsha, Hunan 410011 People’s Republic of China; 2 Medical Research Center, Changsha Central Hospital, 161 Shaoshan Road, Changsha, 410004 Hunan People’s Republic of China; 3 Department of Orthopaedics, Xiangtan Central Hospital, 120 Heping Road, Xiangtan, 411100 Hunan People’s Republic of China

**Keywords:** Secondary syphilis, Total hip arthroplasty, Bone defect, Femoral head, Syphilitic arthritis

## Abstract

**Background:**

*Treponema Pallidum* (TP), the pathogen of syphilis, commonly infects bones in cases of congenital and tertiary syphilis, but it is rare in the primary and secondary stages. With its mild symptoms and rare clinical findings, it might be easy to dismiss the diagnosis of early syphilis. Usually, effective results can be achieved after the conventional strategy of antibiotic treatments, mainly penicillin. To our knowledge, our case is so far the most serious reported case of destructive bone lesion in secondary syphilis, and our treatment for the case is the first strategy using total hip arthroplasty in secondary syphilis.

**Case presentation:**

We present the case of a 71-year-old man with local repeated pain and dysfunction in the right hip. Radiologic examinations showed the disappearance of the ipsilateral femoral head and neck. After excluding the aetiologies of cancer metastasis and tuberculosis, we confirmed the diagnosis of syphilitic arthritis. The patient received the medical treatment of antibiotics and the surgical treatment of total hip arthroplasty. At the follow-up of 1, 3, and 5.5 years after the operation, the patient presented with a pain-free and functional hip prosthesis without local signs of infection and loosening.

**Conclusions:**

This report highlights the difficulties of early diagnosis of secondary syphilis with bone involvement. Bone defect of the femur with secondary syphilis, especially at the proximal femur, was an extremely rare complication in the previous reports. Our case was the first case of a patient who experienced the disappearance of femoral head and neck caused by secondary syphilis. Follow-up after the operation proved the successful treatment of the extensive bone defect of femur by total hip arthroplasty.

## Background

*Treponema Pallidum* (TP), the pathogen of syphilis, has a marked affinity for all organ systems, in which bone tissue is an important lesion position. Destructive bone disease (DBD) is a common complication of congenital and tertiary syphilis; however, DBD, particularly bone defect, rarely occurs in early syphilis [1]. In early syphilis, osteitis or superficial osteolysis is commonly observed and has been reported in a few studies [2–6]. In addition, the lesions are commonly located in the superficial bone, such as the skull, tibia, sternum, clavicle and rib, in the order of highest to lowest incident rate. No previous study has reported the bone defect on the femur in patients with secondary syphilis.

Syphilis comprises both congenital and acquired syphilis. Acquired syphilis is divided into two phases: early syphilis and late syphilis. The former consists of primary syphilis, secondary syphilis and early latent syphilis, while the latter consists of late latent syphilis and tertiary syphilis. A solitary, painless chancre at the inoculation site can be the only symptom of primary syphilis. When the disease progresses to secondary syphilis, generalised mucocutaneous lesions occur in the skin, mucous membranes and lymph nodes. Other tissues and organs can also possibly be involved in this stage. In the latent syphilis, no clinical manifestation exists except the positive results of serologic examinations. The duration of early syphilis is defined as fewer than 2 years, while late syphilis is the presence of the disease for 2 years or more [7]. The diagnosis of syphilitic arthritis not only needs clinical symptoms and laboratory examinations, but also relies on pathologic section. So far, the most accurate methods of diagnosing syphilitic arthritis are dark-field microscope examination and silver stain because both methods can visually observe TP.

The traditional treatment of syphilis using penicillin is sufficient to achieve favourable efficacy. In our case, the patient experienced disappearance of the right femoral head and neck in the secondary stage of syphilis. Thus, the medical treatment of penicillin is insufficient, and the combination of penicillin with a surgical treatment is required. To our knowledge, this case is so far the most serious case of destructive bone lesion in secondary syphilis, and our treatment for the case is the first strategy using total hip arthroplasty (THA) in secondary syphilis.

## Case presentation

**Fig. 1:**
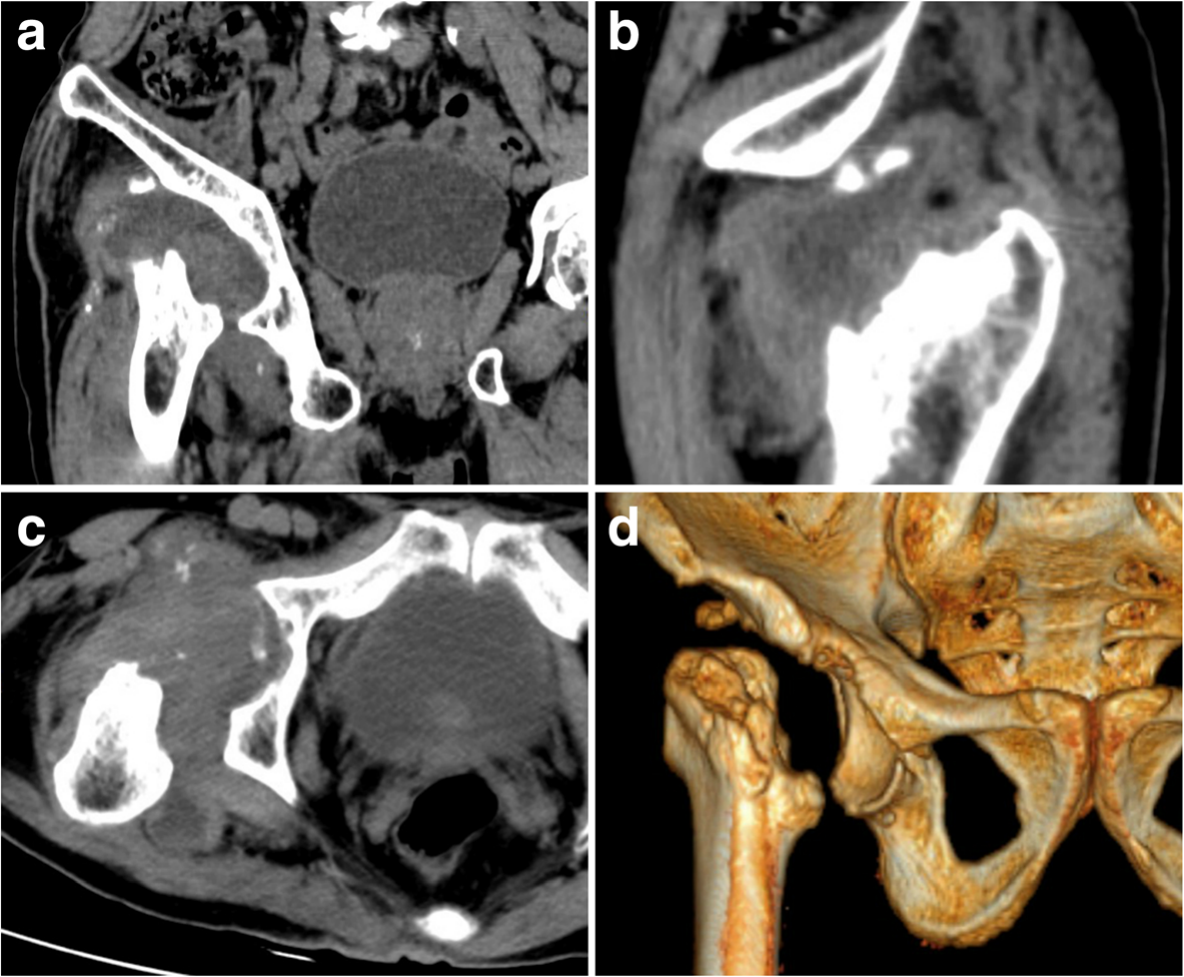
Preoperative computed tomography (CT) scans in the coronal (**a**), sagittal (**b**) and horizontal planes (**c**) with three-dimensional (3D) reconstruction (**d**) shows the normally anatomic structures of the right femoral head and neck disappear

**Fig. 2:**
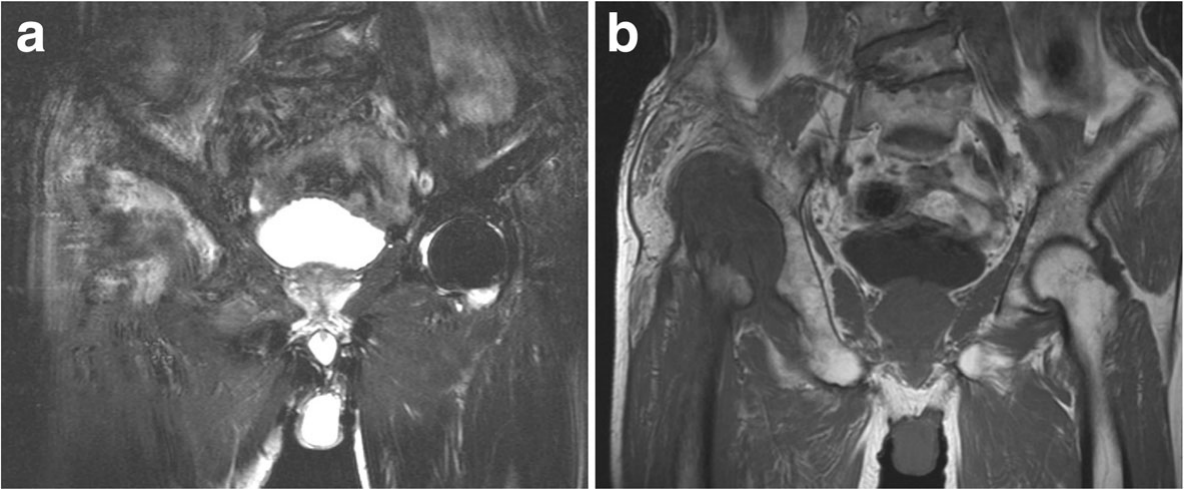
Preoperative T1-weighted (**a**) and T2-weighted (**b**) magnetic resonance image (MRI) shows the original anatomic sites of the right femoral head and neck filled with pathologic tissues

A 71-year-old man was admitted to our hospital with dysfunction in the right hip without a history of hip injury. The patient had local repeated pain over 4 months and gradually lost the capacity to stand and walk because of the hip pain. However, the patient did not report resting pain while sleeping. Before his illness, the patient’s regular activities were walking and going up and down stairs without strenuous exercise. Physical examination revealed tenderness and restricted range of motion of the right hip. Percussion pain was found in the direction of the right lower limb alignment. The length of the right leg was 4 cm shorter than the left leg. Computed tomography (CT) scans with three-dimensional reconstruction showed the deficiency of the right femoral head and neck (Fig. 1). Based on the self-reported clinical history, and the physical and radiologic examination, we firstly presumed that end-stage avascular necrosis of the femoral head, cancer metastasis or tuberculosis (TB) might be the causes of the bone defect. Subsequently, we carried out a series of radiologic and laboratory examinations to validate our judgement. However, in relevant laboratory examinations, including alkaline phosphatase, cancer markers, white blood cell (WBC) count, erythrocyte sedimentation rate (ESR), high-sensitivity C-reactive protein (hs-CRP), TB and purified protein derivative (PPD) test, the results suggested no abnormal values. In addition, magnetic resonance imaging (MRI) and emission computed tomography (ECT) showed a signal change in the right acetabular, suggesting the disappeared femoral head and neck were eroded by pathologic tissue (Fig. 2). Thus, cancer and TB would not be the pathologic cause of the bone defect. Subsequently, the titre of the patient’s rapid plasma regain (RPR) test was 1:128, which indicated the probability of syphilis. The RPR test possesses the advantage of high sensitivity to syphilis and low specificity. Collagenosis, chronic liver diseases, TB and HIV infection may lead to a false-positive result of RPR test by raising the antibody titre. Our patient denied having a history of these diseases. Furthermore, no evidence existed of the related clinical and laboratory findings, such as negative HIV test. The diagnosis of syphilis was eventually validated by the positive result of the *Treponema pallidum* haemagglutination assay (TPHA) test [7, 8], which is highly specific to syphilis. Percutaneous needle biopsy of synovium in the right hip joint also revealed that periangitis, obliterative endarteritis and an intense infiltration of plasma cells accompanied by scattered macrophages and lymphocytes [9, 10] were in the lesion. These correspond to the characteristics of syphilitic lesions. The patient denied having had previous rash or genital ulcers but admitted having unprotected sexual intercourse more than 1 year earlier. Thus, we concluded that syphilitic arthritis caused by early syphilis, rather than cancer metastasis or TB, results in the deficiency of the right femoral head and neck.

To confirm the duration of syphilis, we repeatedly asked the patient his history in detail. The patient admitted that he once sought treatment for pain in the right knee in the Division of Pain Management in our hospital 4 months ago. At that time, the patient reported that the pain in the right knee had lasted for 1 year while the symptom had little influence on the joint function. He had a fever, which peaked at 38.2 °C. The WBC count was 12.5 × 10^9/L. ESR was 72 mm/h. The right lower limb swelled, especially the knee. The puncture synovial fluid culture showed no growth. The values of TB, PPD test and cancer markers were in the normal limits. X-ray film showed osteoarthritis of the right knee joint. Then, the physicians diagnosed osteoarthritis accompanied with active infection, with unknown cause. The patient improved and was discharged after the treatment of painkiller, intravenous mezlocillin, oral meloxicam and the joint injection of sodium hyaluronate.

**Fig. 3:**
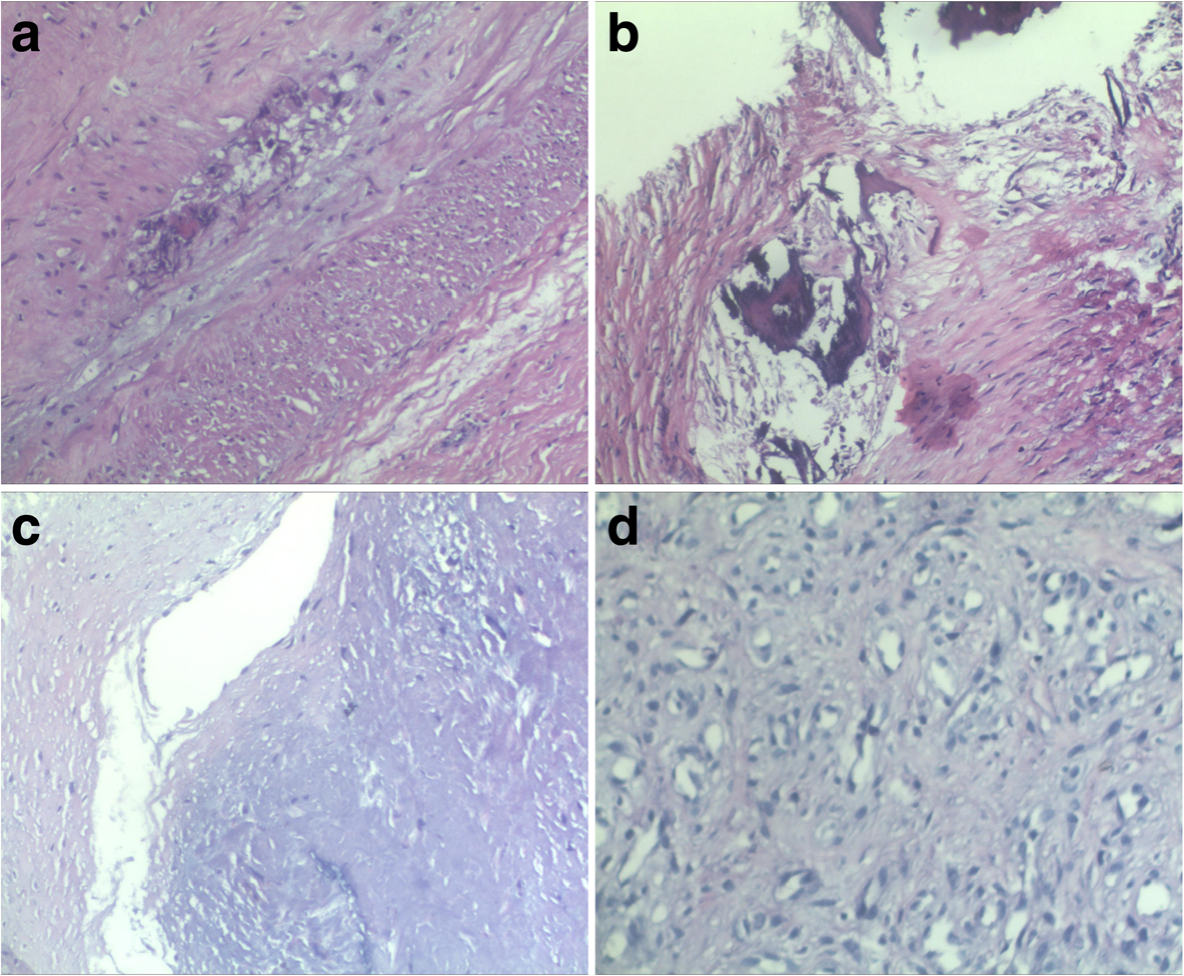
The frozen section examination suggested obliterative endarteritis (**a**), osteonecrosis (**b**), degenerated vessels (**c**) and a perivascular infiltration with plasma cells and lymphocytes (**d**)

After becoming familiarised with the disease course, we planned to perform a THA on the patient, to which the patient consented. The operation was performed in the left recumbent position, under general anaesthesia and through the lateral approach. After opening the joint capsule, clear synovial fluid was discharged and was sampled for bacterial culture. Thickened joint capsule was accompanied by synovial hyperplasia. Extensive bone defect was found on the lateral wall of the right acetabulum. Granulomatous tissue occupied both the acetabular and stump of the femur neck and substituted for the femoral head and neck. No purulent fluid existed. An excisional biopsy was performed from the site of the stump of the femur neck. The frozen section examination suggested that the biological tissue was pathologically modified by syphilis (Fig. 3). The specific features were obliterative endarteritis and a perivascular infiltration with plasma cells and lymphocytes. Furthermore, we observed degenerated vessels and osteonecrosis. After a radical joint debridement, the patient received THA as we originally planned. We performed bone graft to rebuild the extensive acetabular lateral wall defect. The synovial fluid culture showed no growth postoperatively. The paraffin and the frozen sections examinations were consistent.

**Fig. 4:**
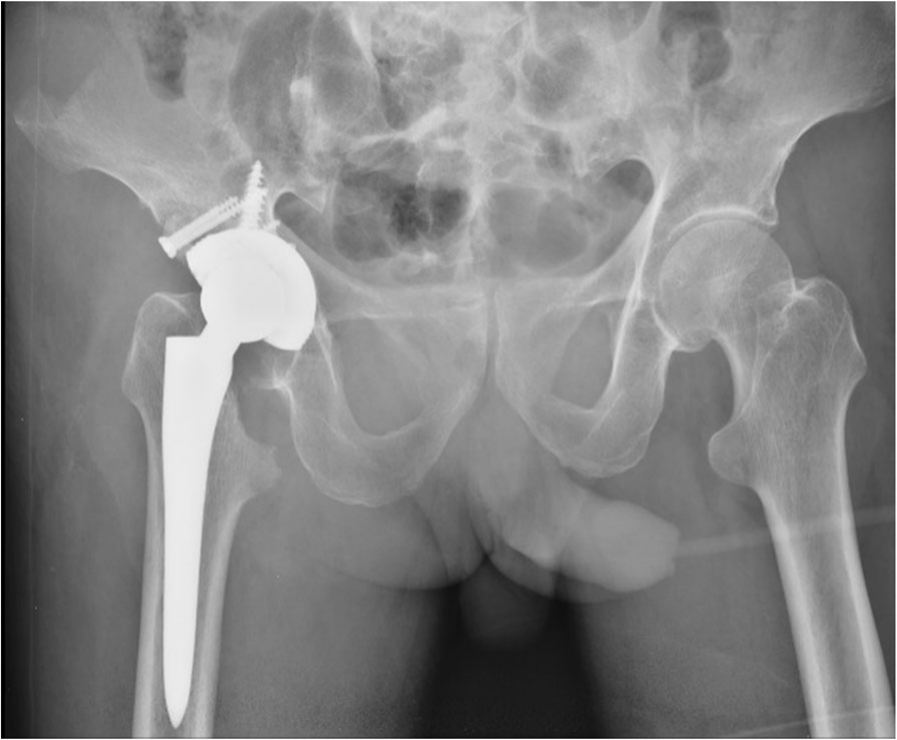
The postoperative x-ray film reveals that the position of the prosthesis is favourable

The intraoperative and postoperative x-ray examinations of the pelvis indicated a favourable implantation of the prosthesis (Fig. 4). The patient underwent standard postoperative physical therapy, including a rehabilitation programme [11, 12]. In addition, the patient received intravenous penicillin 4.8 million units twice daily and azithromycin 0.5 g daily for 12 days and subsequent intramuscular injections of benzathine benzylpenicillin at a dose of 2.4 million units per week for 4 weeks. One year after the last intramuscular injection, an RPR test was performed, and the result suggested a negative finding of syphilis.

**Fig. 5:**
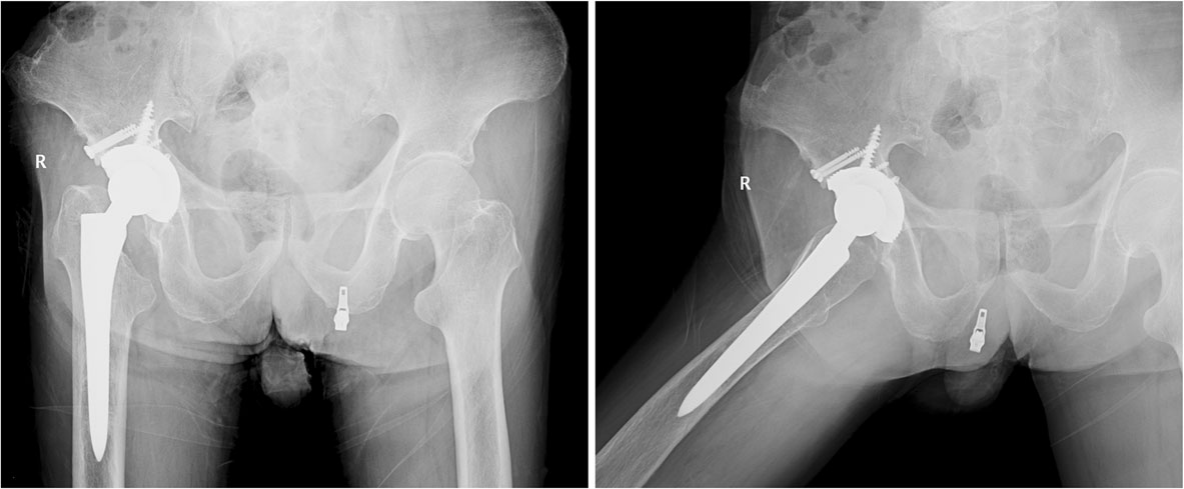
The x-ray examination at the follow-up of 5.5 years after the THA shows no sign of infection and loosening, and bone healed satisfactorily at the bone graft site

We asked the patient to exercise in bed within 3 months after the operation. Afterward, he began weight-bearing exercises. After our treatment, the patient recovered full mobility without pain. At the follow-up of 1, 3 and 5.5 years after the operation, the patient presented with a pain-free and functional hip prosthesis without local signs of infection and loosening. The values of hs-CRP and erythrocyte sedimentation rate (ESR) are also in normal range. X-ray examination revealed the prosthesis location was stable without signs of loosening or migration and that bone healing was satisfactory at the acetabular bone graft site (Fig. 5).

## Discussion and conclusions

We reported a case of the deficiency of unilateral femoral head and neck caused by syphilitic arthritis in secondary stage of syphilis and a successful outcome using the total artificial joint for treating the extensive bone defect. The follow-up at 1, 3 and 5.5 years suggested that THA can achieve a long-term desirable improvement in pain and range of motion. To our knowledge, this is the first case report of a deficiency of the femoral head and neck in secondary stage of syphilis.

We retrospectively analysed the English-language literature from 1967 to the first half of 2017 describing patients with secondary syphilis with involvement of long bone of the limbs (Table 1-2) [6, 13–31]. Twenty-one (78%) patients were male, and 6 (22%) were female. Patients’ median age was 35 years (range, 12–64 years); 22 (81%) patients were 40 years or younger. Ten (37%) patients had HIV infection. Only 14 patients (52%) had rash, 3 patients (11%) had genital ulcer and 7 patients (26%) had generalised lymphadenopathy. Additionally, fever appeared in 9 patients (33%), sweating appeared in 6 patients (22%), loss of appetite appeared in 5 patients (19%), loss of weight appeared in 8 patients (30%) and increased bone pain at night appeared in 10 patients (37%). Considering the symptoms and the clinical manifestations, it is possible to confuse syphilis with cancer metastasis or TB. The median VDRL titre was 1:32 (range, 1:8–1:320), and median RPR titre was 1:128 (range, 1:16–1:512). All the results of the serologic tests of FTA-ABS and MHA-TP/TPHA/TPPA were positive. The total positive rate of the serologic tests was 100%, which demonstrated that the reactive results of Treponemal tests can be instructive to confirm the diagnosis of syphilis. The bone most often affected was the tibia (*n* = 25), followed by fibula (*n* = 11), ulna (*n* = 8), radius (*n* = 5), humerus (*n* = 5) and femur (*n* = 5). In the 5 patients with femur involvement, all the sites of bone lesions occurred in the femur’s lower half. This showed that our patient was the only case of secondary syphilis with involvement of the upper half of the femur in the past 5 decades. Each of the 27 patients underwent at least one of the radiologic or imaging examinations, including x-ray, CT, MRI and bone scan. The detection rate of x-ray for bone involvements was merely 59%. In contrast, MRI/CT and bone scan can detect all bone lesions accurately. Of these, 81% (22 patients) had multifocal bone lesions, and destructive bone lesions were found in 30% of cases. In 8 cases with bone biopsy examinations, plasma cell and/or lymphocyte infiltrations can be observed in 5 patients and the TP detection in 4 patients. All the 27 patients healed after antibiotic treatment. With the exception of the patient who suffered from the pathologic fracture of the left proximal ulna as the complication of bone lesion [6], no other patients received surgical treatment.

**Table 1:** Clinical features in 27 cases of secondary syphilis with long bones of the limbs involvement [6, 13–31]

Parameter	No. (%) of Patients	
Demographic		
Male sex	21/27	(78%)
Age, median (range), year	32	(12–64)
HIV infection	10/27	(37%)
Clinical findings		
Bone pain	27/27	(100%)
Recent history of genital ulcer	3/27	(11%)
Rash	14/27	(52%)
Generalised lymphadenopathy	7/27	(26%)
General manifestations		
Fever	9/27	(33%)
Sweating	6/27	(22%)
Loss of appetite	5/27	(19%)
Loss of weight	8/27	(30%)
Increased bone pain at night	10/27	(37%)
Positive serologic test for syphilis		
Nontreponemal test (Kolmer, VDRL, RPR)	27/27	(100%)
VDRL titre, median (range)^a^	1:32	(1:8–1:320)
RPR titre, median (range)^b^	1:128	(1:16–1:512)
FTA-ABS	10/10	(100%)
MHA-TP/TPHA/TPPA	14/14	(100%)
Sites of affected long bones of the limbs		
Tibia	25/27	(93%)
Fibula	11/27	(41%)
Ulna	8/27	(30%)
Radius	5/27	(19%)
Humerus	5/27	(19%)
Femur	5/27	(19%)
Imaging findings		
Abnormal plain x-ray	13/22	(59%)
Abnormal bone scintigraphy	20/20	(100%)
Abnormal CT or/and MRI	4/4	(100%)
Multifocal bone involvement	22/27	(81%)
Destructive bone lesions	8/27	(30%)
Histologic findings of bone biopsy		
Plasma cell or/and lymphocyte infiltrations	5/8	(63%)
*T. pallidum* detection^c^	4/8	(50%)
Antibiotic treatment		
Benzathine penicillin G	12/27	(44%)
Penicillin G	9/27	(33%)
Procaine penicillin G	4/27	(15%)
Doxycycline	2/27	(7%)
Azithromycin	1/27	(4%)
Cephaloridine	1/27	(4%)
Nafcillin	1/27	(4%)
Ceftriaxone	1/27	(4%)

^a^Determined in 15 patients

^b^Determined in 8 patients

^c^*T. pallidum* was detected by dark-field microscope (*n =* 1), silver stain (*n* = 3)

**Table 2:** Local lesions in 5 cases of secondary syphilis with femur involvement

Authors	Publication year	Lesion sites	Lesion depth
Rosa-Gonçalves et al. [31]	2017	Lower half of the left femur	Cortical bone
Naraghi et al. [22]	2010	The distal right femur	Cortical and subcortical bones
Coyne et al. [29]	2006	Lower half of the right femur	Periostitis
Hansen et al. [26]	1984	The distal both femora	The periosteum and cortex were affected
Siegel et al. [15]	1979	Lower half of the both femora	Cortical bone

TP can multiply and spread inside patients. In the early stage, syphilis commonly presents with slight symptoms and even with no symptoms. In the late stage, the intensive clinical manifestations often lack specific characteristics. The disease can be confused with countless infections and other immune-mediated processes [32]. Due to the clinicians’ incomplete awareness of syphilis, missed diagnosis of syphilis frequently occurred in both early and late stages [33–35]. Even though the diagnosis was finally confirmed, the frustrating process expended a tremendous amount of energy and resulted in a lot of stress for doctors [36, 37]. Therefore, it is not surprising that syphilis gives rise to the moniker, “the great imitator”. For the patient in our case, he originally reported to physicians of the pain in the right knee for 1 year and was transferred to a medical ward for further diagnosis and treatment. The subsequent x-ray examination showed characteristic changes of osteoarthritis, to which the physicians attributed the knee pain. They were unaware that pain symptoms arising from hip disorders frequently radiated to the ipsilateral knee [38–41]. The slight pain of the hip lesion was concealed. In terms of the patient’s fever during his hospital stay, the synovial fluid of the right knee, at which the syphilitic arthritis was erroneously localised by the physicians, was sampled for bacterial culture, and no growth was found. The physicians ignored the fact that even though TP existed in the sample, TP cannot be cultured and has the limits of direct visualisation [42]. Another clue for the presence of syphilis was neglected. The diagnosis of syphilis was finally missed after the fever abated with treatment of intravenous mezlocillin. Because the patient came to our hospital on foot at the first hospitalisation, as opposed to by wheelchair at the second hospitalization, if the bone lesion had been detected early, the normal structure of the right hip joint would have been almost preserved by using the regular penicillin treatment, thus avoiding THA. Thus, early diagnosis of syphilis is required for prompt treatment of bone destruction. Low- and middle-income countries (LMICs) and high-income countries (HICs) have obvious differences in terms of control strategies of syphilis [43]. In LMICs, syphilis is detected only in suspected patients or prenatal care testing. However, in North America and Western Europe, syphilis testing is listed as one of the screening methods in all infectious diseases to detect the infection early. Therefore, the early and correct diagnosis of syphilis can be usually made in the HICs owing to the widespread testing of syphilis. In our patient, our key point is that the early diagnosis was made based on the clue of a positive result of RPR test, which was routinely included in preoperative examinations. We argue that routine screening of a RPR test may help expedite diagnosis, and most importantly, the incidence of missed diagnosis of syphilis will dramatically decrease by means of this test method.

Bone tissues are not commonly involved focal sites in syphilis, especially in the early stage. As a result, bone lesions are often ignored in patients with early syphilis [3]. In the largest case series to date, Reynolds and Wasserman included and analysed approximately 10,000 patients given a diagnosis of early syphilis over 21 years from 1919 to 1940 [44]. Only 15 (0.15%) patients were found to have destructive bone disease. In another retrospective study of 854 patients with secondary syphilis, the incidence of destructive bone disease was only 0.2% (2 patients) [45]. In 1952, another radiologic survey found seven cases (9%) of skull involvement in 80 patients with secondary syphilis [46]. An increasing trend of bone involvement in syphilis was found in recent studies [3, 4, 30, 47–55]. Some experts demonstrated that the incidence of syphilis-caused destructive bone lesions in previous survey may be underestimated [26]. This might be due to the manifestation of syphilis and the limitation of techniques: 1) the slight pain and asymptomatic lesion fail to draw doctors’ and patients’ attention; 2) previous radiologic techniques cannot detect a large part of bone lesions. With the advance of radiologic technology in recent decades, such as CT and MRI, syphilis-caused destructive bone lesions are increasingly observed year after year [54]. Thus, the detection of bone involvement should not be ignored if syphilis is identified. Bone lesions in the initial stage of syphilitic arthritis are characterised by osteitis and osteolysis that may not induce noticeable signal change in x-ray examinations. For patients with syphilis, either in the early or advanced stages, CT and MRI may be additionally applied if the patients have slight pain at a body site because these imaging techniques may enhance the detection rate of bone involvement, especially the minimal change.

Bone lesions in syphilis are mainly involved in the skull, tibia, sternum, clavicle and rib [3, 5, 26, 55–57]. Our retrospective analysis also verifies that the tibia is the most frequently affected long bone in the limbs. The superficial bones seem to be more susceptible to TP invasion than deep bones. Rare reports referred to syphilis-caused lesions in the femur and hip joint [26, 34]. When secondary syphilis influences the skeletal structures, periostitis, osteitis, osteomyelitis and osteolysis are the major pathologic changes of syphilis-caused bone lesion, and destructive bone lesions rarely occur. In our review, of 27 patients with secondary syphilitic bone diseases, only 8 (30%) patients had destructive bone lesions. TP invades periosteal vascular beds by hematogenous spread, resulting in periostitis and granulation tissue formation. The extension of inflammation into Haversian canals induces osteitis and osteomyelitis. As the disease progresses, osteoblastic activity can be affected. If osteogenesis is not enough to compensate for osteolysis, destructive bone lesions occur. We reviewed the English-language literature describing patients with secondary, tertiary and congenital syphilis accompanied with the destructive bone lesions in syphilitic hip arthritis (Table 3). We found only four related cases (one patient with secondary syphilis, three patients with tertiary syphilis) [34, 58–60]. Of these, two cases represented the treatments for the destructive bone lesions in syphilitic hip arthritis. Owing to the limited extent of destructive bone lesions, both patients underwent standard medical treatments without surgical treatments. For the patient in our case, only 1 year and 4 months separated the initially mild pain to the dysfunction of the right hip that resulted from the erosive destruction of the femoral head and neck. Such a surprising rate and severity of lesion progression have never been observed and reported before. This case demonstrates that the bone destruction caused by TP can exceed the expected severity. The bone erosion progressed so rapid that THA had to be applied to reconstruct the joint to recover the patient’s mobility.

**Table 3:** The treatments in four cases of tertiary and congenital syphilis with syphilitic hip arthritis

Authors	Stage of syphilis	Publication year	Lesion sites	Lesion depth	Medical treatment	Surgical treatment
Spyridonidis et al. [34]	Tertiary syphilis	2002	Left hip joint	Osteolysis and periosteal reaction	Intravenous penicillin G (16 million units per day) for 5 days; intramuscular benzyl-penicillin (2.4 million units a week) for 3 weeks	None
Brain et al. [58]	Congenital syphilis	1926	Both hip joints	Erosion at the inner end of the epiphyseal line of the femur	Not given	Not given
Coblentz et al. [59]	Congenital syphilis	1970	Right hip joint	Destructive metaphysitis	Not given	Not given
Greenall et al. [60]	Congenital syphilis	2010	Both hip joints	Metaphysitis and bony destructive changes	Benzylpenicillin and Cefotaxime	None

Although the primary lesion of the right hip joint was infected with TP, the preoperative results of ESR, CRP and WBC count were in the normal limits, which means no existence of active infection. The risk of postoperative infection was so small that the patient can receive surgical treatment as long as the surgeons eradicated the source of infection during the operation. Long-term follow-up showed no indication of infection and bone defect, which proved this procedure successful.

Although the importance of rapid recovery protocols has been emphasised in recent years [61], the activity recommendations should be individualised for each patient [61, 62]. In our patient, considering his life expectancy, the uncemented extensive porous-coated components were chosen as the artificial hip joint prosthesis. In addition to the occurrences of extensive bone destruction, the rapid recovery protocol was not deemed to be applicable to him. We performed the well-recognized physical exercise programs [11, 12, 63], namely additive interventions, including straight leg raises and unilateral resistance training of the quadriceps and hip-abductor muscles in the early postoperative phase and weight-bearing exercises in the late phase. Starting from the weight-bearing exercises, the patient’s activity gradually increased to the general level such as walking and going up and down stairs. Usually, too little activity leads to decreased bone destiny, which results in early loosening, and too much activity leads to increased wear, which results in late loosening. From the angle of individualised evaluation, we were convinced that our patient’s current amount of exercise achieves a balance point between bone density and wear [62]. The evidence was that no sign of loosening and bone defect were detected from the follow-up x-ray film, the conventional mainstay in evaluating THA compared to CT and MRI [64]. In our patient, although there is no indication of early loosening (before 10 years), late loosening, which commonly occurs after 10 years postoperatively, is still uncertain. Thus, the long-term effects of THA remain to be seen.

In conclusion, we reported a unique case of bone defect in the femur and hip joint in secondary stage of syphilis. Bone involvement should not be ignored for syphilis even in the early stage, and early diagnosis of syphilis is required for avoiding advanced bone destruction. Moreover, THA can achieve successful long-term efficacy of desirable full motion in the affected joint. 

## Abbreviations

3D: Three-dimensional
CT: Computed tomography
DBD: Destructive bone disease
ECT: Emission computed tomography
ESR: Erythrocyte sedimentation rate
HICs: High-income countries
hs-CRP: High-sensitivity C-reactive protein
LMICs: Low- and middle-income countries
MRI: Magnetic resonance imaging
PPD: Purified protein derivative
RPR: Rapid plasma regain
TB: Tuberculosis
THA: Total hip arthroplasty
TP: *Treponema Pallidum*
TPHA: *Treponema pallidum* haemagglutination assay
WBC: White blood cell
